# Structural Models of Human eEF1A1 and eEF1A2 Reveal Two Distinct Surface Clusters of Sequence Variation and Potential Differences in Phosphorylation

**DOI:** 10.1371/journal.pone.0006315

**Published:** 2009-07-28

**Authors:** Dinesh C. Soares, Paul N. Barlow, Helen J. Newbery, David J. Porteous, Catherine M. Abbott

**Affiliations:** 1 Medical Genetics Section, Molecular Medicine Centre, Institute of Genetics and Molecular Medicine, Western General Hospital, University of Edinburgh, Edinburgh, United Kingdom; 2 School of Chemistry, University of Edinburgh, Edinburgh, United Kingdom; 3 Centre for Translational and Chemical Biology, University of Edinburgh, Edinburgh, United Kingdom; Institute of Infectious Disease and Molecular Medicine, South Africa

## Abstract

**Background:**

Despite sharing 92% sequence identity, paralogous human translation elongation factor 1 alpha-1 (eEF1A1) and elongation factor 1 alpha-2 (eEF1A2) have different but overlapping functional profiles. This may reflect the differential requirements of the cell-types in which they are expressed and is consistent with complex roles for these proteins that extend beyond delivery of tRNA to the ribosome.

**Methodology/Principal Findings:**

To investigate the structural basis of these functional differences, we created and validated comparative three-dimensional (3-D) models of eEF1A1 and eEF1A2 on the basis of the crystal structure of homologous eEF1A from yeast. The spatial location of amino acid residues that vary between the two proteins was thereby pinpointed, and their surface electrostatic and lipophilic properties were compared. None of the variations amongst buried amino acid residues are judged likely to have a major structural effect on the protein fold, or to affect domain-domain interactions. Nearly all the variant surface-exposed amino acid residues lie on one face of the protein, in two proximal but distinct sub-clusters. The result of previously performed mutagenesis in yeast may be interpreted as confirming the importance of one of these clusters in actin-bundling and filament disorganization. Interestingly, some variant residues lie in close proximity to, and in a few cases show differences in interactions with, residues previously inferred to be directly involved in binding GTP/GDP, eEF1Bα and aminoacyl-tRNA. Additional sequence-based predictions, in conjunction with the 3-D models, reveal likely differences in phosphorylation sites that could reconcile some of the functional differences between the two proteins.

**Conclusions:**

The revelation and putative functional assignment of two distinct sub-clusters on the surface of the protein models should enable rational site-directed mutagenesis, including homologous reverse-substitution experiments, to map surface binding patches onto these proteins. The predicted variant-specific phosphorylation sites also provide a basis for experimental verification by mutagenesis. The models provide a structural framework for interpretation of the resulting functional analysis.

## Introduction

Translation elongation factor alpha (eEF1A) has a pivotal role in protein synthesis, since it is responsible for delivering aminoacylated tRNAs to the A site of the ribosome. In higher vertebrates, eEF1A is found in two variant forms, encoded by distinct genes [1], and with different expression patterns. The near-ubiquitous form, eEF1A1, is expressed in all tissues throughout development but is absent in adult muscle and heart [2], [3]. The latter tissues express instead eEF1A2 as do certain other cell types including, notably, large motor neurons, islet cells in the pancreas and enteroendocrine cells in the gut [4].

**Figure 1:**
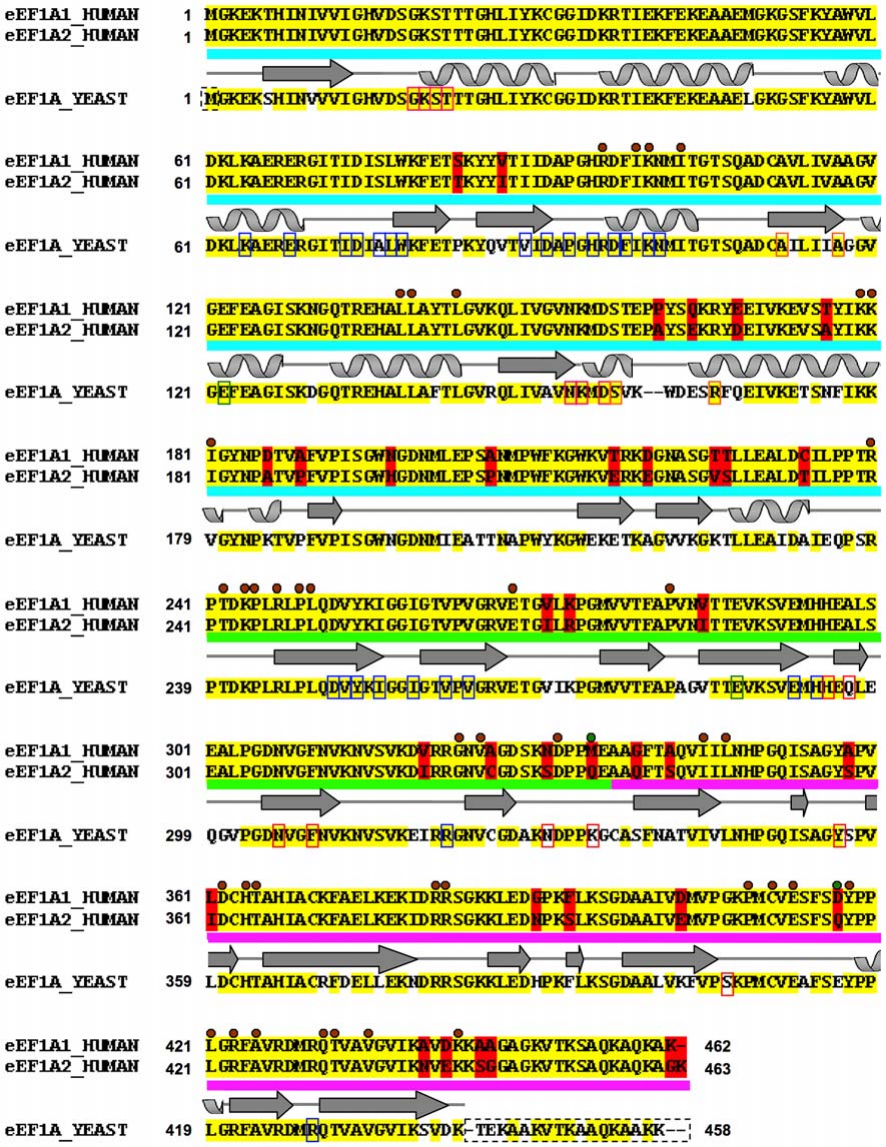
Sequence alignment between human eEF1A1 and eEF1A2 and yeast template. The pair-wise sequence alignment between human eEF1A1 and eEF1A2 is shown: identical residues (yellow background), variant residues (red background). The aligned yeast eEF1A template is shown below with identical residues to the human sequences highlighted (yellow background) and any variant position between yeast and either human sequence shown with a white background. The two human sequences share 92% sequence identity with each other and each show ∼81% sequence identity with the yeast protein. The domain boundaries (domain I: cyan; domain II: green; domain III: pink), and STRIDE [44] secondary structure assignment is traced above the yeast template sequence (arrows = beta-strands; coils = alpha-helices). The amino acid residues involved in domain-domain contacts are indicated with a brown circle (green circle for non-identical equivalent residues between two human variants); those involved in the binding of C-terminal fragment eEF1Bα are indicated on the yeast sequence with blue rectangles; residues involved in GDP-binding indicated in pink rectangles; and those disordered in the yeast crystal structure are indicated with a dashed rectangle. Yeast mutagenesis data and motifs are highlighted on its sequence: mutations involved in actin bundling/disorganization (red rectangles) [70], [71]; mutations that affect translational fidelity (green rectangles) [77]; mutations that reduce dependence on eEF1B (orange rectangles) [78], [79].

Despite sharing 92% sequence identity (Figure 1), paralogous human eEF1A1 and eEF1A2 have different functional profiles. They exhibit similar translation activities, but have different relative affinities for GTP and GDP [5]. eEF1A1 binds GTP more strongly than GDP, whereas the opposite is the case for eEF1A2. The GDP dissociation rate constant is seven-fold higher for eEF1A1 than for eEF1A2, and the GDP/GTP preference ratio is 0.82 for eEF1A1, but 1.50 for eEF1A2. Surprisingly, since this would predict its greater reliance on GTP-exchange factors, eEF1A2 appears to show little or no affinity for the components of the guanine-nucleotide exchange factor (GEF) complex eEF1B in yeast-two-hybrid experiments [6]. eEF1B is made up of three subunits, eEF1Bα, eEF1BΔ (called eEF1Bβ in plants) and eEF2Bγ [7]. The eEF1Bα and eEF1BΔ subunits possess guanine nucleotide-exchange activity, whereas the eEF2Bγ subunit is thought to have a more structural role, tethering the complex to the membrane of the endoplasmic reticulum.

eEF1A1 has been implicated in additional non-canonical functions (reviewed in [8]), including actin-binding and bundling [9], apoptosis [10], nuclear transport [11], proteasomal-mediated degradation of damaged proteins [12], heat shock [13] and transformation [14]. eEF1A2 has been less extensively studied at the biochemical level, so it is not yet clear how many of these non-canonical functions are shared by this variant. For example, eEF1A2 has been shown to have a role in actin remodeling in cells [15], but has not been shown directly to bind to actin. In humans, eEF1A2 has been shown to have oncogenic properties when inappropriately overexpressed, and has been implicated in ovarian, breast, pancreatic, liver and lung cancer [16], [17], [18], [19], [20], although the mechanism for overexpression remains elusive, and no mutations have been identified in ovarian tumors [21]. Loss of expression of eEF1A2, on the other hand, has been shown in mice to result in motor neuron degeneration reminiscent of motor neuron disease, or amyotrophic lateral sclerosis [3], [22], [23].

There appears to be a complex interplay between eEF1A and its binding partners that has the net effect of balancing its canonical activity in peptide synthesis with its non-canonical actin-binding and bundling functions. Such a balance may be critical for an as yet little-understood integration of gene expression and cytoskeletal dynamics. In higher eukaryotes, different cell types are likely to have different requirements in terms of both protein synthetic capacity and cytoskeletal regulation. It is possible that competition for binding between aminoacyl-tRNA and actin may tilt the balance between the two functions. The presence of the two variants in mammals creates the potential for greater complexity than is seen in yeast: whilst *Saccharomyces cerevisiae* has two genes encoding eEF1A and *Schizosaccharomyces pombe* has three, the encoded proteins are identical within a given species [24], [25]. One hypothesis is that the two mammalian variants eEF1A1 and eEF1A2 differ in the extent to which they participate in peptide synthesis versus actin bundling, and that this lies behind their differential expression in various cell types. For example, motor neurons express eEF1A2 and not eEF1A1 [4]; these cells can reach up to a meter in length in humans, and it is tempting to speculate that their cytoskeletal organization would have different constraints from those of, say, hepatocytes. This hypothesis predicts that there will be differences in binding affinities or specificities for aminoacyl-tRNA and/or actin mediated by differences in the amino acid residues that comprise the respective binding sites.

From the pair-wise sequence alignment between the two human variants (Figure 1), it is apparent that many of the changes involve substitution of Ser or Thr (total of 11); although no Tyr amino acid residues are lost or gained. This observation leads to a second hypothesis; that differential phosphorylation of the two variants effectively amplifies their chemical differences and promotes functional divergence. It is well known that all four subunits (*i.e.* one monomeric eEF1A subunit and three eEF1B subunits) of the assembled ‘heavy’ elongation factor complex are targets for kinases. Large-scale proteomics studies revealed that conserved Tyr residues (Tyr29, Tyr85, Tyr86, Tyr141, Tyr162, Tyr254) in both human eEF1A variants [26], [27], [28], [29] are phosphorylated. In most studies, it is impossible to judge whether peptides variously identified as originating from eEF1A1 or eEF1A2 are actually specific to one variant, as the peptides identified are from regions that are completely conserved between eEF1A1 and eEF1A2. Rikova et al. [27] identify eEF1A2 as a substrate for anaplastic lymphoma kinase in lung cancer patients. This is consistent with expression data as this variant is overexpressed in lung adenocarcinoma cell lines [20] while the *EEF1A2* gene is amplified in lung cancer cell lines [30]. Furthermore, we have shown that whilst eEF1A2 is overexpressed in a significant subset of primary lung tumors, eEF1A1 expression remains constant when compared with normal lung tissue (J.Boyd, W.Wallace and C.Abbott, unpublished data). On the other hand, Molina et al. [28], in a study of phosphopeptides in human embryonic kidney cells, identified phosphorylation of, unequivocally, eEF1A1 at Tyr29 and Ser163. Rush et al. [26] identified a number of tyrosine phosphorylation sites in eEF1A from various cell lines, but in each case the phosphorylated peptides are from completely conserved regions and thus could have originated from either variant. Additional confirmation of tyrosine phosphorylation was presented by Panasyuk et al. [31] who proposed that while both variants could be involved in phosphotyrosine-mediated processes, eEF1A2 had greater potential to participate in such signaling pathways. Lamberti et al. confirmed serine and threonine phosphorylation of eEF1A, and suggested other likely Ser and Thr phosphorylation sites based on a bioinformatics study of eEF1A1 [32]. However all of their predicted sites (Ser18, Ser157, Ser316, Ser383, Thr242 and Thr432) are conserved between the two variants and their study did not distinguish between the two variant forms. In agreement with one of the abovementioned phosphorylation predictions, Eckhardt et al. confirmed phosphorylation of Thr432 in eEF1A1 via mass spectrometry and site-directed mutagenesis [33]. Thus the hypothesis that non-conserved Thr and Ser residues of eEF1A1 and eEF1A2 are targets for phosphorylation remains untested. One way to begin investigating this hypothesis is to adopt a structural approach (in conjunction with sequence-based phosphorylation predictors) and to examine whether the residues in question are exposed and accessible to kinases or buried and inaccessible.

To date there have been no published mutagenesis reports specifically aimed at delineating binding sites in the human eEF1A variants but there are mutagenesis data, and several three-dimensional (3-D) structures, for yeast eEF1A. We therefore set out to construct and validate 3-D models of human eEF1A1 and eEF1A2 on the basis of homology with a known structure of yeast eEF1A. Modeling by homology is a well-established technique, and protein models have wide-ranging applications in biomedical research (see [34] for recent review). The plan was to examine the locations in the structure of the non-conserved residues, to assess the extent to which these are exposed (in the case of Ser or Thr) for possible phosphorylation, or occupy putative ligand and protein-binding sites, and thereby to test the aforementioned hypotheses.

## Methods

### Target sequences, template identification and selection

The target sequences used were eEF1A1 [residues 1-443 (out of 462); SwissProt Accession No: P68104] and eEF1A2 [residues 1-443 (out of 463); SwissProt Accession No: Q05639]. A BLAST search [35] for each of the target sequences against the Protein Data Bank (PDB) [36] returned six highly similar potential templates [PDB IDs: 1F60, 1G7C, 1IJE, 1IJF, 2B7B, 2B7C] from *Saccharomyces cerevisiae* [37], [38], [39] with an E-value of 0.0 (sequence identity ∼81%). All these structures were solved in complex with the C-terminal eEF1Bα fragment and in some cases with GDP, GDPNP, or GDP-Mg^2+^ bound. Upon superposition, the structural arrangements/orientations for the three domains are almost identical (Cα RMSD: 0.1 to 0.7 Å) among the six structures (Supplementary file S1). The highest resolved structure from this set – the 1.67-Å X-ray-derived eEF1A protein structure from yeast [38] (PDB ID: 1F60, chain A) was selected as the template.

### Target-Template alignments

Optimal template selection, and target-to-template alignment is key to the success of any modeling exercise [40], [41]. The alignment between the targets and template sequences (Figure 1) for modeling purposes, was based on a multiple-sequence alignment among them using the program ClustalX [42]. Because of their high sequence similarity, the alignment was predictably trivial, and no further manual editing was required. The targets eEF1A1 and eEF1A2 share 81% and 80% sequence identity (∼89% similarity) respectively with the template (Figure 1). Unsurprisingly, PSIPRED v2.5 [43] predicted near-identical secondary structure for both variants (not shown). The STRIDE [44] identified secondary structure for the template when overlaid on the sequence alignment, showed that the two gaps were indeed placed within a loop region (Figure 1).

### Model building and refinement

The two target-template alignments were individually used as inputs for the program Modeller release 8 version 2 [45]. Twenty models were generated and, in each case, the ones with the lowest value of the *objective function score* were selected as the representative models. Non-identical side-chain residues for each representative model were optimized using the side-chain replacement program, SCWRL version 3 [46], [47]. The models were then protonated under SYBYL version 6.9 (Tripos Associates, St. Louis, MO, USA), and subject to brief energy minimization (20 steps steepest descent, followed by 20 steps conjugate gradients) employing the Tripos forcefield [48] under SYBYL v6.9 to remove clashes and bad geometries.

### Model evaluation

The models were checked for valid stereochemistry (Supplementary file S2) using PROCHECK version 3.5.4 [49]. The coarse packing quality of the models was assessed using the WHAT IF server (http://swift.cmbi.ru.nl/servers/html/index.html) [50], [51], and the models additionally evaluated using the MetaMQAP II server (https://genesilico.pl/toolkit/unimod?method=MetaMQAPII) [52]. The final models are available for download (Supplementary file S3).

### Analysis of model properties

PyMol (http://www.pymol.org; DeLano Scientific, San Carlos, CA, USA) was used for structure visualization. Yeast eEF1A-eEF1Bα interacting amino acid residues and eEF1A domain-domain contacts (Figure 1) were identified using the Protein Interactions Calculator (PIC) [53]. PIC identifies all hydrophobic interactions within 5 Å, main-chain to main-chain, main-chain to side-chain and side-chain to side-chain hydrogen bonds, ionic interactions, aromatic-aromatic interactions, aromatic-sulfur interactions, and cation-pi interactions. Proximity of putative binding site residues with respect to variant amino acid residues was identified by using a 5 Å sphere radius from the binding site amino acid residues under PyMol. Structural superpositions were undertaken using MultiProt (http://bioinfo3d.cs.tau.ac.il/MultiProt/) [54]. Solvent-accessibility calculations were performed using GETAREA version 1.1 (http://curie.utmb.edu/getarea.html) using a sphere probe radius of 1.4 Å [55]. Electrostatic surface representations were generated using GRASP [56] and lipophilic surface renditions created using MOLCAD [57] under SYBYL v6.9.

### Phosphorylation prediction

The human eEF1A1 and eEF1A2 amino acid sequences were used to search the translated nucleotide database ‘nr/nt’ at NCBI using BLAST (tblastn) (http://www.ncbi.nlm.nih.gov/blast/Blast.cgi) [35]. The retrieved orthologues were then used as input to ClustalX for multiple sequence alignment. BOXSHADE was used for shading sequence conservation. Phosphorylation site prediction was performed using the NetPhos version 2 server (http://www.cbs.dtu.dk/services/NetPhos/) [58] and mapped onto the alignment and 3-D model.

## Results and Discussion

### Quality assessment of the 3-D models of human eEF1A1 and eEF1A2

The models of eEF1A1 and eEF1A2 were created based upon the yeast crystal structure of eEF1A and evaluated for stereochemistry, packing quality and expected accuracy. The Ramachandran dihedral statistics [49], [59] for both models were good; eEF1A1: 93.6% most favored, 5.1% additionally allowed, 1.1% generously allowed, 0.3% disallowed; and eEF1A2: 92% most favored, 6.7% additionally allowed, 1.3% generously allowed, 0% disallowed. Additionally, the packing quality [50], [51] for the models attained overall average quality control scores of −0.89 (eEF1A1) and −0.87 (eEF1A2). To place this in context, incorrect models give scores of<−3.0; lower quality models<−2.0; and the average quality of 200 highly refined X-ray structures −0.5 (+or −0.4). The absolute global deviations, expressed as Root Mean Square Deviation (RMSD) and Global Distance Test Total Score (GDT_TS [60]) by MetaMQAP II [52] for the models versus the unknown true structures were sound (eEF1A1: GDT_TS: 80.3, RMSD: 1.9 Å and eEF1A2: GDT_TS: 78.5; RMSD: 2.0 Å), indicating high quality models. These evaluation statistics are in line with the high sequence identity (∼81%) and similarity (89%) of the targets to the eukaryotic yeast template, the presence of only two gaps in the target-template alignment (Figure 1), and the high-resolution quality of the determined template crystal structure (1.67 Å) [38]. Taken together, the result is that the quality of the models created approaches that of experimentally determined structures [40].

Another, albeit less similar template from the archaeon, *Sulfolobus solfataricus* [61], [62] (∼53% sequence identity) was also detected from the BLAST search [35] against the PDB [36] and has been used as a template in previous modeling studies for the human variants [32], [63]. This template was not included in our modeling protocol because of its significantly lower sequence similarity, and larger number of gaps (3%) when compared with the yeast template. Moreover, comparison of the secondary structure of this potential template (PDB ID: 1JNY chain A) with the eukaryotic yeast template (PDB ID: 1F60 chain A) reveals differences – for example, two beta-strands at positions 212–223 in yeast that encompass two variant amino acid residues in humans, are absent in archaea; the loop after the third alpha-helix in domain I in the archaeal structures [61], [62] contain eleven residues (Arg66-Phe76) that are disordered - five of these residues are directly involved in eEF1Bα-binding. This would translate into a dearth of distance restraints for that region for modeling purposes. Additionally, the orientation of two helices and the switch 1 region are different [61], [62]. Finally, while their individual domains overlay reasonably well (Cα RMSD: 1 to 1.9 Å), the relative orientation of domain I with respect to domains II and III in yeast and archaea is different [62]. This may or may not be attributable to the structural rearrangements that accompany eEF1Bα-binding in the yeast structure, since the domain-domain orientations adopted by a ligand-free yeast eEF1A, is at yet unknown. Nonetheless, given the abovementioned limitations of using the archaeal template, it is prudent to adopt the ligand-bound eEF1A yeast template, singularly, for modeling other eukaryotic targets.

### Domain-domain contacts between the two variants are conserved

**Figure 2:**
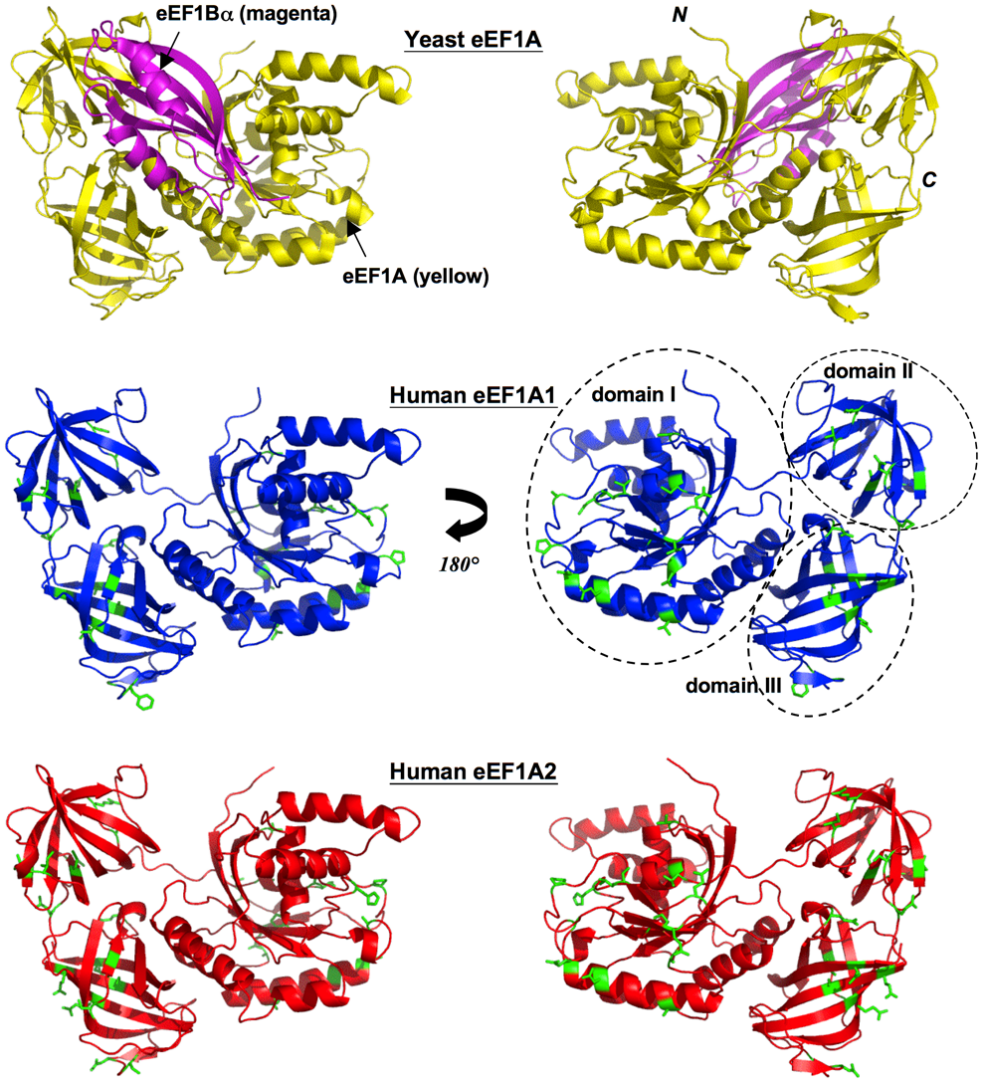
Yeast template, human eEF1A1 and eEF1A2 models. Two views rotated by 180° about the *y*-axis depicting cartoon schematic representations of: the yeast eEF1A (yellow)-eEF1Bα C-terminal fragment (magenta) crystal structure (top panel). The 3-D models of eEF1A1 (blue, middle panel), and eEF1A2 (red, lower panel) show the location of variant side-chains (in stick representation) between the two proteins, colored green. Secondary structure elements have been assigned by default settings in PyMol (http://www.pymol.org) and position of domains labeled.

Structurally, each model (like the template) consists of three domains, referred to as domain I, domain II and domain III (Figure 2). Domain I (residues 1-to-240) is made up of a Rossmann-fold topology. Domains II (residues 241-to-336) and III (residues 337-to-443) are made up almost entirely from beta-strands; each domain contains two beta-sheets that form a beta-barrel.

The 32 out of 36 variations in amino acid residues between the two human proteins are distributed over all three domains (Figures 1 and 2). The remaining four variations were not modeled because they are present within a disordered region at the C-terminus of the yeast template, and hence it is unknown how they may influence the overall structure or binding-interactions. Only six (out of 32) amino acid residues that are variable between the two human proteins are completely buried in the modeled structures. Two of these are conservative changes (Val87Ile and Leu361Ile) while two others entail substitution of an alanine for another small residue (Ala326Cys and Ala342Ser); the remaining pair of substitutions (Pro161Ala, Ala189Pro), while not so obviously conservative, can readily be accommodated within the protein core as judged from their excellent packing scores. Thus there is nothing to suggest that individual domain structures will differ between the two human variants.

**Figure 3:**
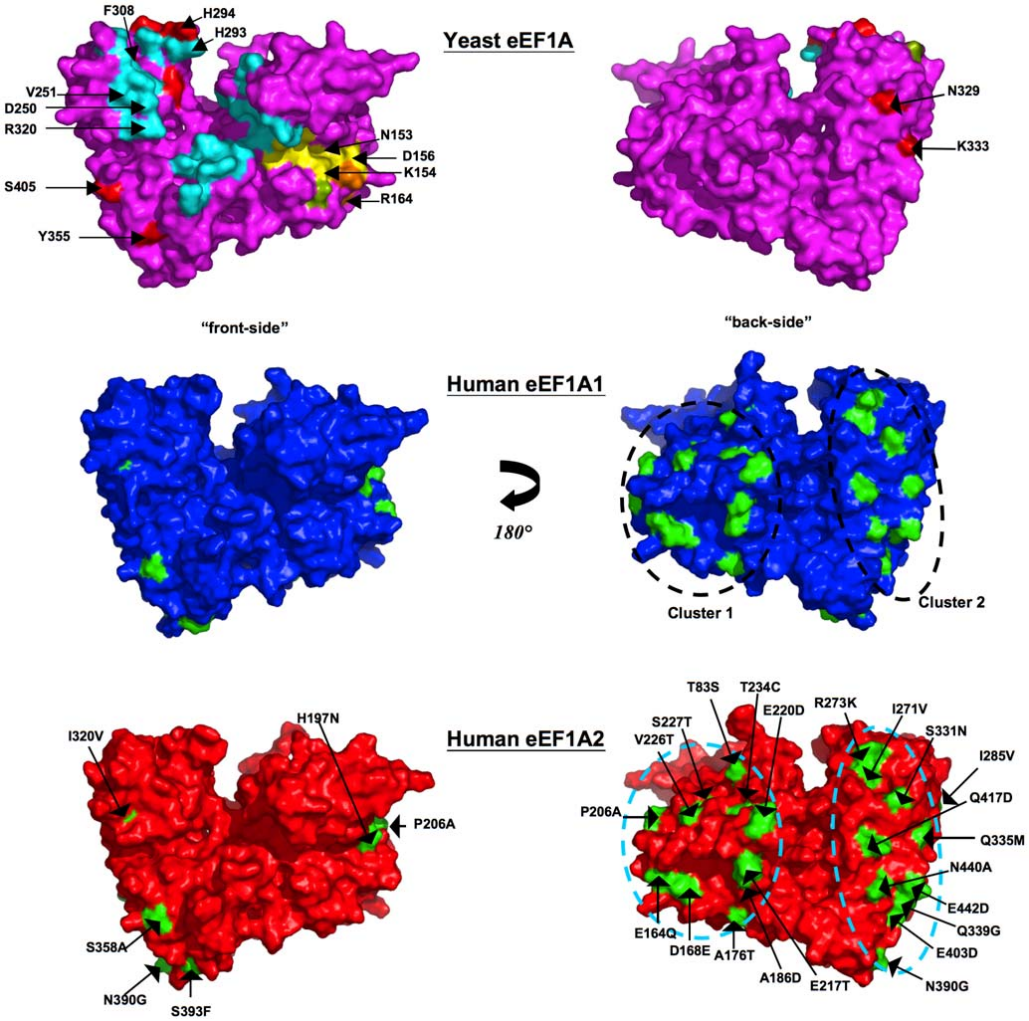
Location of variations in amino acids mapped onto surface. Two equivalent views rotated by 180° about the *y*-axis depicting a surface rendition of the yeast eEF1A crystal structure colored magenta (top panel), and the 3-D models of eEF1A1 colored blue (middle panel) and eEF1A2 colored red (bottom panel). Locations of exposed variant side-chains are mapped onto the surface of the two model proteins (colored green) and labeled on the eEF1A2 model - the variant residue from eEF1A1 is shown on the right-hand side of the label. The two sub-clusters are apparent in this representation. The location of the C-terminal eEF1Bα-binding site (cyan) [38] and GDP-binding site (yellow) [39] is mapped on the crystal structure. Also highlighted (red) on its surface are: mutations that reduce actin disorganization induced by overexpression of eEF1A, inhibit actin-bundling without altering translation *in vivo*, and reduce actin-bundling [70], [71]. There are no variants in proximity to those residues implicated on the basis of mutagenesis to be involved in translational fidelity (green) [77]. However, two variant positions in humans - Gln164Glu and Glu168Asp are in close proximity to Arg166 – a conservative mutation for the equivalent residue in yeast (Arg164Lys) was shown to reduce dependence on eEF1B (orange) [78], [79]. Gln164Glu and Glu168Asp, however, both retain their main-chain to main-chain H-bonds with Arg166. Note: for clarity the three proposed aminoacyl-tRNA-binding residues [38] are not shown, since they overlap with already highlighted positions implicated in binding eEF1Bα (His293 and Arg320) and actin (His294).

A total of 40 out of 42 residues involved in domain-domain contacts amongst the three domains are identical (Figure 1) for the two variants and the yeast template (contacts identified using PIC [53]). The exceptions are Met/Gln335Lys (residue in linker connecting domains II and III) and Asp/Gln417Glu (domain III) in human eEF1A1/eEF1A2, versus yeast eEF1A. The side-chains of these amino acid residues are, however, largely surface-exposed (Figure 3) and they retain their inter-domain H-bonds (from their main-chain oxygen atoms to the side-chains of Cys409 and Lys242, respectively). This excellent conservation of inter-domain interfaces reinforces the inference from the very high quality of the homology-based models that both human variants may adopt the same conformation as observed in the eEF1Bα-complexed structure of yeast eEF1A. These observations do not shed light on the issue of whether or not domain rearrangements accompany association or disassociation of eEF1B. It is noteworthy that of the 42 residues involved in domain-domain contacts within the eEF1Bα-bound yeast structure, 17 are not absolutely conserved in the archaeal *Sulfolobus solfataricus* sequence; likewise, of the 40 residues involved in domain-domain contacts within the unbound archaeal structure, 14 are not absolutely conserved in the yeast sequence (Supplementary file S4). This observation is consistent with the notion that the domain arrangement within the archaeal EF1A structure is not necessarily mirrored in the eEF1B-free forms of human eEF1A, and reinforces our decision to model human eEF1A1 and eEF1A2 on the yeast template.

### Surface-exposed variant amino acids between the two proteins lie in sub-clusters located on one-side of the protein

**Figure 4:**
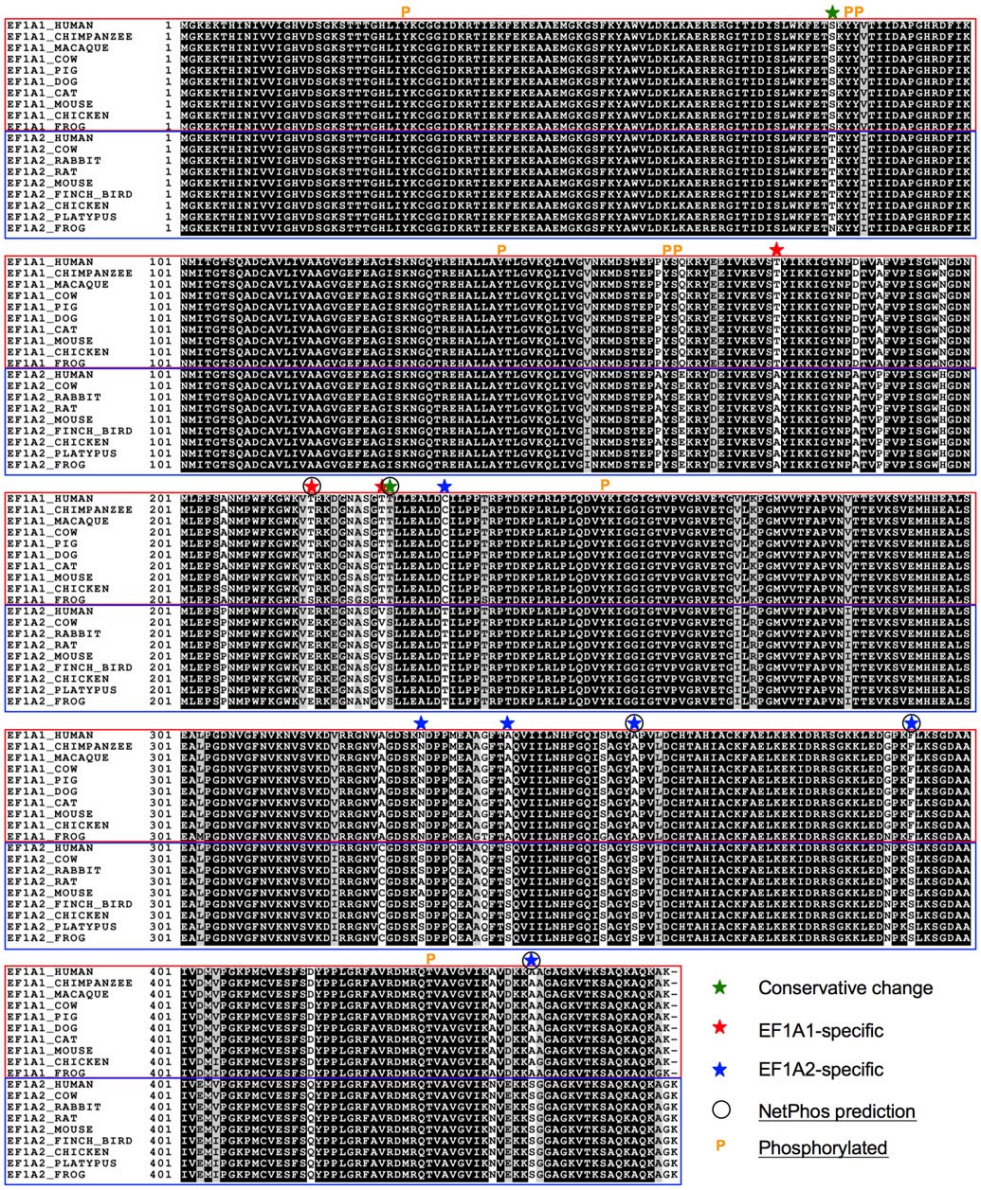
Multiple sequence alignment of eEF1A1 and eEF1A2 orthologues. ClustalX alignment of eEF1A1 and eEF1A2 sequences from a range of higher order eukaryotes. The results are shaded using BOXSHADE v3.21 (black background = strictly conserved; grey or white background = conservatively substituted or non-conserved). A star-symbol denotes the position of variant Ser and Thr amino acid residues for the two proteins and color-coded according to variant (red = eEF1A1-specific; blue = eEF1A2-specific). NetPhos-predicted phosphorylation sites are indicated by a circle, and experimentally determined phosphorylation sites shown with a ‘P’ symbol (these are mapped on the models in Supplementary file S6).

Mapping the variable residues on the model surfaces (middle and bottom panel, Figure 3) reveals that almost all of them congregate on one face of the molecule – on the opposite face from the C-terminal eEF1Bα-binding site (top panel, Figure 3) as proposed in [6] on the basis of the yeast crystal structure. These non-conserved residues are located in two distinct sub-clusters on the same face, but separated by a distance of ∼27 Å (measured between residues 220 and 417); a circular band of residues lying within domain I (cluster 1, 12 residues, ∼35 Å in diameter), and a swathe of residues spread across domains II and III (cluster 2, 14 residues, ∼46×12 Å). It seems highly unlikely that such clustering would occur by chance and it is therefore reasonable to infer that the clusters correspond to binding sites for one or more partners. Such an inference is reinforced by multiple- sequence alignments with eukaryotic orthologues in which it is apparent that the residues contributing to these clusters are very highly conserved within their respective eEF1A1 and eEF1A2 families (Figure 4).

Other models for eEF1A1 have been created previously. Lamberti et al. [32] created a homology model of the eEF1A1 variant, based on *Sulfolobus solfataricus* EF1A, which is probably a less suitable template than yeast eEF1A for several reasons discussed previously. Marco et al. [64] used a model based on the yeast eEF1A template to assess the ability of a potent inhibitor of protein synthesis (didemnin B) to bind human eEF1A1. Neither of these studies extended to human eEF1A2. The only comparative study between eEF1A1 and eEF1A2 to date was performed by Kanibolotsky et al. [63] who reported near-identical modeled structures and speculated on differences in the conformational dynamics of the two variants, but did not highlight the sub-clustering of variable amino acid residues that is a striking feature of the current models. The ligands for these putative binding sites, and any consequent differences in binding specificities of eEF1A1 versus eEF1A2, are a matter of speculation.

### Comparison of electrostatic and lipophilic surface properties

**Figure 5:**
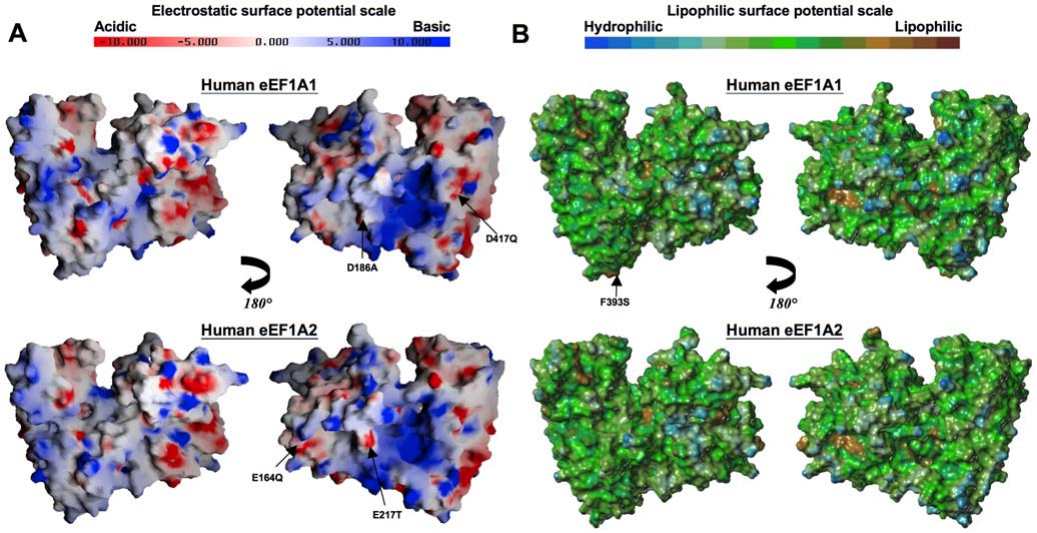
Surface properties of the models eEF1A1 and eEF1A2. (A) Two equivalent views, rotated by 180° about the *y*-axis, of a GRASP-generated [56] surface electrostatic representation of eEF1A1 (upper panel) and eEF1A2 (lower panel). Negative charge is colored red and positive charge colored blue, ranging from -10 kT to +10 kT (k = Boltzmann's constant; T = temperature in Kelvin). Charged residues not present in either protein (non-conservative charged substitutions only) are labeled – the variant equivalent residue is shown on the right-hand side of the label. (B) Two equivalent views, rotated by 180° about the *y*-axis, of a MOLCAD-generated [57] lipophilic surface rendition of the models. Regions of high lipophilicity or hydrophobicity are colored brown and regions of high hydrophilicity are colored blue.

A closer inspection of surface properties of the modeled structures should help understand how variations in sequence mediate functional differences between eEF1A1 and eEF1A2. Although virtually all the variable residues appear as two sub-clusters on one face of the molecule (Figure 3), this face has similar overall electrostatic and lipophilic characteristics in both molecules (Figure 5). This is consistent with the fact that conservative substitutions of residues account for three-quarters of the variation between the two proteins (Figure 1). Individual conservative changes likely alter functional properties only to a small degree. But the combined effects on molecular recognition of numerous conservative variations congregated in a surface patch could be more dramatic. To this may be added the influence of the few non-conservative variations; for example, the eEF1A2 variant (in comparison with eEF1A1) has replaced neutral polar residues with electronegative residues at positions Glu164 and Glu217, while negative residues are substituted with Ala186 and Gln417 (in cluster 1); the replacement of Phe393 (in eEF1A1) with Ser393 (in eEF1A2) is a particularly notable substitution (in cluster 2).

### Analysis of variant amino acid residues with respect to putative protein/ligand binding sites

#### (a) eEF1Bα

The yeast eEF1A structure used as a template in the current study was solved in complex with a fragment of eEF1Bα [38]. Complex formation between yeast eEF1A and eEF1Bα (fragment) (Figure 2) buries ∼3558 Å^2^ of surface area [38]. The binding site on eEF1A for the eEF1Bα C-terminal fragment lies mainly on domains I and II, with only a single contact with domain III (Arg428) [38]. A total of 24 out of 26 eEF1A residues that form the binding site for eEF1Bα, including all eight residues that participate in salt-bridge formation, are invariant in yeast and both human proteins (Figure 1). Two interface residues, Ala76 and Val89 in yeast, are substituted in both human proteins by similarly sized residues, Ser76 and Ile89, respectively. Thus there are no differences between the two human versions of eEF1A in a highly conserved eEF1Bα-binding site. The only variant (between eEF1A1 and eEF1A2) amino acid residue within proximity of the eEF1Bα-binding site is Val/Ile320 that lies within 5 Å of three interface residues - Asp252, Val253 and Arg322. Val320 (eEF1A1) and Ile320 (eEF1A2) are involved in a hydrophobic interaction with Val253. Hence we may conclude that all three proteins engage with eEF1Bα C-terminal fragment in the same way and are likely to have similar affinities, despite yeast-two-hybrid studies that failed to demonstrate an eEF1A2-eEF1B interaction [6]. Mansilla et al suggest that there may be brain-specific variants of eEF1Bα, and although one has been described in human [65], we have shown that this appears to result from expression of a processed pseudogene with no orthologue in mice and is thus unlikely to be of functional significance [66]. However, a further yeast-two-hybrid screen using mouse eEF1A2 as bait for a mouse brain cDNA library also failed to pull out any eEF1B subunits [67]. One explanation for the negative yeast-two-hybrid results is that the N-terminal segment of eEF1Bα (>100 amino acid residues) - which was not present in the yeast crystal structure - wraps around and contacts the “far-side” of eEF1A1 [6], stabilized by direct contacts at these sites of variation. Moreover, it is known that eEF1A1 also interacts with eEF1BΔ [6]; eEF1BΔ shows sequence similarity to eEF1Bα, and can also function as a GEF [7]. Clearly, further experiments assessing binding between eEF1A2 and eEF1B subunits using methodologies other than yeast-two-hybrid screens are needed.

#### (b) aminoacyl-tRNA

The placement of aminoacyl-tRNA in the A site of the ribosome is catalyzed by eEF1A. The equivalent residues in the human variants of eEF1A that have been proposed to be part of the aminoacyl-tRNA binding site [38] are Arg322, His295 and His296, which lie within domain II. These residues are conserved in both human variants. As mentioned above, Arg322 is also involved in eEF1Bα–binding, and hence the fact that Val/Ile320 lies within 5 Å of Arg322 might have a bearing on aminoacyl-tRNA binding.

#### (c) GDP/GTP

The co-crystal structures of yeast eEF1A bound to GDP, GDPnP (a non-hydrolyzable analogue of GTP) and GDP-Mg^2+^ [37], [39] provided a detailed picture of the guanine-binding pocket. This work revealed the critical importance of Gly19, Lys20, Ser21, Thr22, Asn153, Lys154 and Asp156 (all in domain I) within the binding sites for these ligands (Supplementary file S5). These observations were in agreement with previously performed mutagenesis studies [68] – for example, the Asn153Thr and Asp156Asn mutations of yeast eEF1A resulted in dramatic reduction in translational fidelity. Interestingly, between the unbound eEF1A-eEF1Bα and the eEF1A-eEF1Bα–GDP/GDPnP bound conformations, Asp156 is reoriented so as to form H-bonds to the guanine-base [39]. This guanine-binding pocket is absolutely conserved in both human variants and such an observation must be reconciled with their differences in GDP/GTP preference ratios. In this respect it may be relevant that some variant residues - Gln164Glu, Asn197His and Ala206Pro (eEF1A1 versus eEF1A2 – equivalent to Glu162, Asn195 and Thr204, respectively, in yeast) lie close to the guanine-binding pocket. Of particular note is residue 197 that lies immediately adjacent to Asp156; in the eEF1A1 model, as in the yeast eEF1A structure, there is a H-bond between the Asn197 (Asn195 in yeast) and Asp156 side-chains. Such an H-bond cannot exist in eEF1A2 (in which position 197 is occupied by a His), although it could be replaced by an ionic interaction. Such a situation in which a key GDP/GTP contact residue is perturbed could lie behind the differential guanine binding of the two variants.

#### (d) Actin

Actin and aminoacyl-tRNA-binding to eEF1A are mutually exclusive [69]. Previously undertaken mutagenesis studies in yeast [70], [71] identified up to eight residues in eEF1A clustered within domains II and III, which (i) reduce actin disorganization induced by overexpression of eEF1A in yeast, (ii) inhibit actin-bundling without altering translation *in vivo*, and/or (iii) reduce actin-bundling. Two such residues implicated in actin-related functions correspond to variant residues for the human proteins within sub-cluster 2. Indeed, a site-directed mutation in yeast eEF1A, Asn329Ser, fortuitously corresponds to changing eEF1A1 Asn331 to its eEF1A2 equivalent, Ser331. This is one of two mutations (along with Asn329Asp) that were shown in yeast to reduce actin-bundling or actin disorganization that is induced by overexpression of eEF1A [70], [71]. Additionally, another equivalent yeast eEF1A residue that is implicated in actin-related functions is variable (Met335Gln) between the two human proteins in sub-cluster 2. Also of note is that yeast eEF1A Phe308 is implicated in actin-related functions by mutagenesis [71]; the equivalent in humans (Phe310) contacts Ala326 in eEF1A1 (hydrophobic interaction) or Cys326 in eEF1A2 (aromatic-sulfur interaction). In a similar vein, yeast eEF1A Tyr355 is critical for actin-related functions and the side-chain of its human equivalent (Tyr357) is adjacent to the side-chain of either Ala358 in eEF1A1 or Ser358 in eEF1A2. So these differences (Ala326Cys and Ala358Ser) between the two human variants could have ramifications for adjacent sites critical for actin-related functionality. Previously performed mutagenesis studies thus serendipitously confirm the importance of one of the two clusters/binding patches in actin-related functions; this suggests that the two variants have different actin-binding and bundling properties consistent with our original hypothesis. Moreover, this study provides a direction for further mutagenesis experiments that should target sub-cluster 2 as a means of understanding the role of the actin-eEF1A interaction.

### Homologous (reverse) substitution mutagenesis can reconcile functional disparities

Our observation of two clusters of variant residues on the surface of eEF1A1/2 creates the possibility of conducting rational homologous (reverse) substitution mutagenesis. This is a well-established approach to delineating precisely which amino acid residues are responsible for specific functional differences between highly similar proteins e.g. [72], [73]. Thus, the involvement of those residues predicted to lie within or close to the actin-binding cluster Asn/Ser331, Met/Gln335, Ala/Cys326 and Ala/Ser358 should be investigated by this route. Similarly, eEF1A1 mutations Asn197His (potentially important for GTP/GDP-binding), and Val320Ile (may be involved in aminoacyl-tRNA-binding and eEF1Bα-binding) should be probed.

### Using the modeled structures to infer differences in potential sites of phosphorylation

Many of the amino acid differences between the two human variants involve substitution of Ser or Thr residues (total of 11) and it is interesting to observe the presence and almost strict conservation of variant-specific Ser and Thr positions among orthologues in higher eukaryotes (Figure 4). Four out of the eight experimentally confirmed phosphorylation sites for the human variants are not conserved in yeast. A sequence-based NetPhos phosphorylation analysis of the two variants predicts (>0.5 probability score) five of these eleven sites to be both variant-specific and potential phosphorylatable. These include Thr217 and Thr227 in eEF1A1; and Ser358, Ser393 and Ser445 in eEF1A2. The last two residues in eEF1A2 have >0.95 probability scores [Ser393 (0.99) and Ser445 (0.96)]. When a kinase encounters a potential substrate for phosphorylation, it recognises the surface of the protein. From the 3-D models, Thr217, Thr227 (eEF1A1), Ser358 and Ser393 (eEF1A2) all expose their hydroxyl-groups (Figure 3; Supplementary file S6). Ser445 (eEF1A2) on the other hand, lies in the C-terminal region that was not modeled; the equivalent region in the yeast crystal structure was disordered. It is known that phosphorylation frequently occurs in disordered regions [74], hence this site too forms another prime candidate for phosphorylation.

Although overexpression of eEF1A2 has been implicated in a range of different tumor types, no such role for eEF1A1 has yet been established. It is tempting to speculate that any difference in oncogenic potential between the two variants lies in the different phosphorylation potential of eEF1A1 and eEF1A2. It will thus be important to confirm phosphorylation of the two most likely eEF1A2 sites - Ser393 and Ser445 - using, for example, mass spectrometry. Furthermore, it would be useful to create phosphorylation mimics, through mutagenesis of Ser or Thr to Asp or Glu [75], [76], or to a non-phosphorylatable amino acid residue such as the eEF1A1-equivalent. If Ser393 and Ser445 are indeed phosphorylated, it would be interesting to raise antibodies to the phosphorylated forms of eEF1A2 and use these to establish whether any differences can be seen between eEF1A2 expressed in tumor compared to normal tissue. Such studies would not only yield biological insights but could also provide useful diagnostic reagents.

### Conclusions

We set out to use homology modeling to address two hypotheses: first, amino acid differences between the two variants dictate differences in relative binding affinities or specificities for aminoacyl-tRNA and/or actin, reflecting differential roles in various cell types; second, differential phosphorylation of the two variants effectively amplifies their chemical differences and promotes functional divergence. The 3-D models reveal two distinct sub-clusters of sequence variation on one face of the proteins. We observed that variable amino acid residues within one of these clusters overlapped with residues implicated in actin-bundling and disorganization. Some other variable residues participate in interactions with, or lie in close proximity to, amino acid residues directly involved in binding GTP/GDP, eEF1Bα and aminoacyl-tRNA. These findings are in predictive agreement with our first hypothesis and identify, high-priority targets for rational site-directed mutagenesis including homologous reverse substitution experiments. In addition the models, in support of our second hypothesis, suggest possible differences of phosphorylation and hence future experiments to investigate differences in phosphorylation between the variants.

## Supporting Information

File S1 Yeast crystal structure comparison. (A) Table depicting the six different yeast eEF1A crystal structures in the Protein Data Bank (PDB) [4], resolution, PDB chains and solved residue lengths, along with each of their corresponding interacting eEF1Bα proteins and ligands. (B) MultiProt [5], [6] structural superposition of all six eEF1A crystal structures highlights the structural conservation among them. Each structure color-coded differently. (C) Table depicting the pair-wise structural comparisons for all vs. all yeast crystal structures. The Combinatorial Extension [7] calculated Cα root mean square deviation (RMSD) is shown in Angstroms along with the structural alignment length between the two proteins. The closeness of the structures is evident in this table.
(0.13 MB PDF)

File S2 Ramachandran evaluation plots for models of human eEF1A1 and eEF1A2. Top: Ramachandran plot scores for eEF1A1: most favored regions: 93.6%; additional allowed regions: 5.1%; generously allowed regions: 1.1%; disallowed regions: 0.3%. Bottom: Ramachandran plot scores for eEF1A2: most favored regions: 92%; additional allowed regions: 6.7%; generously allowed regions: 1.3%; disallowed regions: 0%. Ideally, one would hope to have>90% residues in the “most favored” regions of the Ramachandran plot [1], [2].
(0.09 MB PDF)

File S3 3-D model co-ordinates of human eEF1A1 and eEF1A2. Note: co-ordinates for both models (human eEF1A1 and human eEF1A2) are provided in one PDB file.
(1.13 MB TXT)

File S4 Sequence alignment between archaeal EF1A (*Sulfolobus solfataricus*) and yeast (*Saccharomyces cerevisiae*) eEF1A. Pair-wise sequence alignment between eEF1A from yeast vs. EF1A from archaea. From the structures of yeast and archaea, residues involved in domain-domain contacts are depicted as follows: *  =  yeast amino acid residue involved in domain-domain contact, when bound to eEF1Bα; &  =  archaea amino acid residue involved in domain-domain contact, free of eEF1Bα; underlined residues  =  not present in the crystal structures. Identical positions in the alignment are shown with a yellow background; variable positions involved in a domain-domain contact are highlighted with a red background. A total of 42 residues are involved in domain-domain contacts in the eEF1Bα-bound yeast structure, 17 are non-identical at the equivalent position in the archaeal sequence, and conversely, there are 40 residues involved in domain-domain contacts within the unbound archaeal structure, 14 of which are non-identical in the yeast sequence.
(0.08 MB PDF)

File S5 Guanine-binding pocket in yeast and human variants. Close-up equivalent views of residues involved in binding of GTP/GDP: the yeast template (top left); all six yeast eEF1A structures superposed (top right) and colored differently (see their corresponding PDB IDs below); human eEF1A1 (bottom left); and human eEF1A2 (bottom right). Labeled residues include: Gly19, Lys20, Ser21, Thr22, Asn153, Lys154, Asp156 [GTP/GDP-binding residues] and Asn195 (yeast), Asn/His197 (human eEF1A1/eEF1A2) that show presence of H-bond (indicated by a yellow dashed line and distance in Angstroms) with Asp156, absent in human eEF1A2. Atomic color scheme for yeast template and human variants: nitrogen: blue; oxygen: red; carbon: yellow (note: for clarity hydrogen atoms not shown).
(0.15 MB PDF)

File S6 Location of known and variable potential phosphorylatable residues on eEF1A1 and eEF1A2 models. Cartoon schematic representation of the 3-D models of eEF1A1 and eEF1A2 depicting the location of the known (orange) and potential (green) phospho-Ser, -Thr and -Tyr residues (side-chain shown in stick representation without hydrogen atoms, for clarity). Potentially phosphorylated Ser445 in eEF1A2 is located in a disordered region, and is hence not seen in the figure. Note: all experimentally confirmed phosphorylation sites are conserved between the human variants, and have not been unambiguously determined specifically to each or both variants. For purposes of the figure, phosphorylated residues are depicted on the models as reported in the PhosphoSitePlus database (http://www.phosphosite.org) [1]: for eEF1A1 (Tyr29, Tyr85, Tyr86, Tyr141, Tyr162, Ser163, and Thr432); and for eEF1A2 (Tyr29, Tyr141, and Tyr254).
(0.54 MB PDF)
