# Supplementary material for: Structural Models of Human eEF1A1 and eEF1A2 Reveal Two Distinct Surface Clusters of Sequence Variation and Potential Differences in Phosphorylation
Source: PLoS One. 2009 Jul 28;4(7):e6315. doi: 10.1371/journal.pone.0006315 (PMC2712093; doi:10.1371/journal.pone.0006315)
Supplement: File S1 — Yeast crystal structure comparison. (A) Table depicting the six different yeast eEF1A crystal structures in the Protein Data Bank (PDB) [4], resolution, PDB chains and solved residue lengths, along with each of their corresponding interacting eEF1Bα proteins and ligands. (B) MultiProt [5], [6] structural superposition of all six eEF1A crystal structures highlights the structural conservation among them. Each structure color-coded differently. (C) Table depicting the pair-wise structural comparisons for all vs. all yeast crystal structures. The Combinatorial Extension [7] calculated Cα root mean square deviation (RMSD) is shown in Angstroms along with the structural alignment length between the two proteins. The closeness of the structures is evident in this table. (0.13 MB PDF) [file pone.0006315.s001.pdf]

Supplementary file S1: Yeast crystal structure comparison.

(A)

PDB ID	Reference	Resolution	eEF1A	eEF1Bα	Ligand
1F60	[1]	1.67 Å	A: 2-441	B: 1117-1206	-
1G7C	[2]	2.05 Å	A: 4-443	B: 1117-1206	GDPnP
1IJE	[2]	2.4 Å	A: 4-441	B: 1117-1206	GDP
1IJF	[2]	3.0 Å	A: 2-441	B: 1117-1206	GDP-Mg^{2+}
2B7B	[3]	2.6 Å	A: 2-441	B: 1117-1206 (K205A)	GDP
2B7C	[3]	1.8 Å	A: 5-441	B: 1117-1206 (K205A)	-

(B)

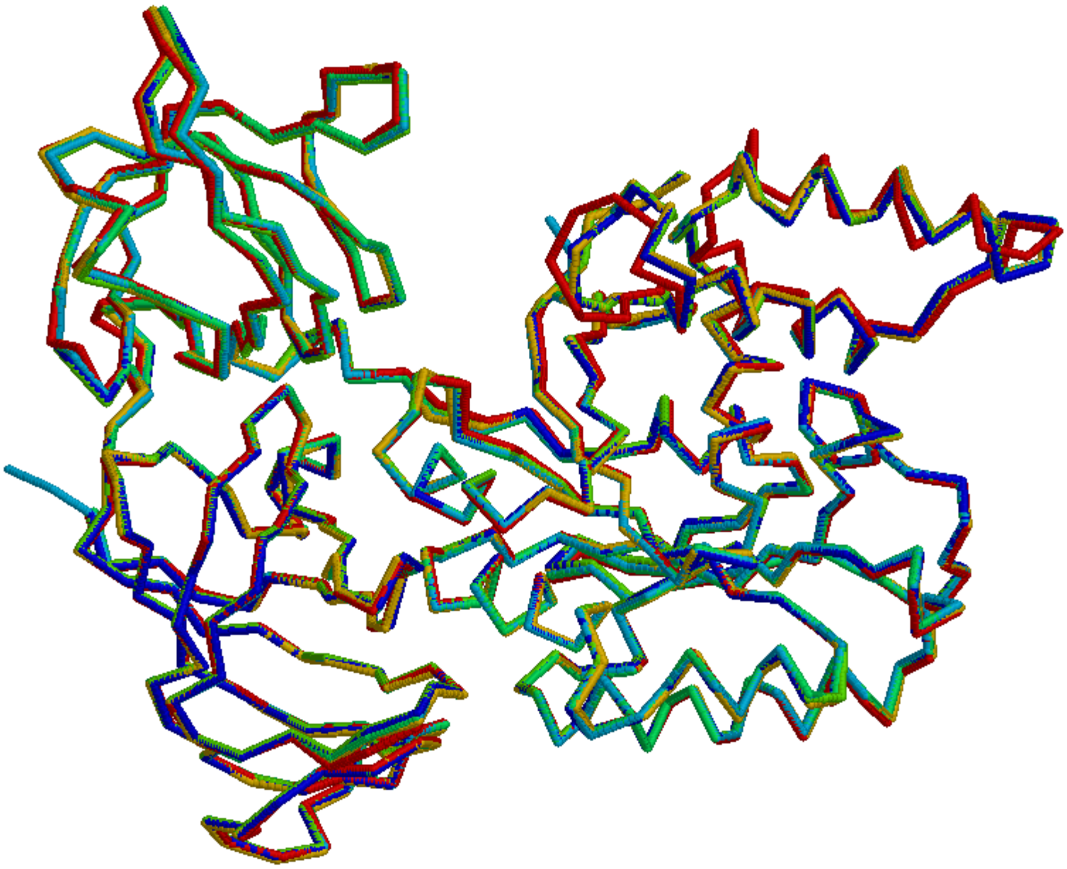

(C)

**PDB ID**	**1F60**	**1G7C**	**1IJE**	**1IJF**	**2B7B**	**2B7C**
**1F60**						
**1G7C**	0.26 Å (438 aa)					
**1IJE**	0.27 Å (438 aa)	0.18 Å (438 aa)				
**1IJF**	0.13 Å (440 aa)	0.29 Å (438 aa)	0.30 Å (438 aa)			
**2B7B**	0.29 Å (440 aa)	0.33 Å (438 aa)	0.31 Å (438 aa)	0.32 Å (440 aa)		
**2B7C**	0.69 Å (437 aa)	0.68 Å (437 aa)	0.68 Å (437 aa)	0.72 Å (437 aa)	0.70 Å (437 aa)	

(A) Table depicting the six different yeast eEF1A crystal structures in the Protein Data Bank (PDB) [4], resolution, PDB chains and solved residue lengths, along with each of their corresponding interacting eEF1Bα proteins and ligands. **(B)** MultiProt [5,6] structural superposition of all six eEF1A crystal structures highlights the structural conservation among them. Each structure color-coded differently. **(C)** Table depicting the pair-wise structural comparisons for all vs. all yeast crystal structures. The Combinatorial Extension [7] calculated Cα root mean square deviation (RMSD) is shown in Angstroms Å along with the structural alignment length between the two proteins. The closeness of the structures is evident in this table.

References:

1. Andersen GR, Pedersen L, Valente L, Chatterjee I, Kinzy TG, et al. (2000) Structural basis for nucleotide exchange and competition with tRNA in the yeast elongation factor complex eEF1A:eEF1Balpha. Mol Cell 6: 1261-1266.
2. Andersen GR, Valente L, Pedersen L, Kinzy TG, Nyborg J (2001) Crystal structures of nucleotide exchange intermediates in the eEF1A-eEF1Balpha complex. Nat Struct Biol 8: 531-534.

3. Pittman YR, Valente L, Jeppesen MG, Andersen GR, Patel S, et al. (2006) Mg2+ and a key lysine modulate exchange activity of eukaryotic translation elongation factor 1B alpha. J Biol Chem 281: 19457-19468.
4. Berman HM, Westbrook J, Feng Z, Gilliland G, Bhat TN, et al. (2000) The Protein Data Bank. Nucleic Acids Res 28: 235-242.
5. Shatsky M, Nussinov R, Wolfson HJ (2002) MultiProt - A multiple protein structural alignment algorithm. Algorithms in Bioinformatics, Proceedings 2452: 235-250.
6. Shatsky M, Nussinov R, Wolfson HJ (2004) A method for simultaneous alignment of multiple protein structures. Proteins-Structure Function and Bioinformatics 56: 143-156.
7. Shindyalov IN, Bourne PE (1998) Protein structure alignment by incremental combinatorial extension (CE) of the optimal path. Protein Eng 11: 739-747.
