# Supplementary material for: Structural Models of Human eEF1A1 and eEF1A2 Reveal Two Distinct Surface Clusters of Sequence Variation and Potential Differences in Phosphorylation
Source: PLoS One. 2009 Jul 28;4(7):e6315. doi: 10.1371/journal.pone.0006315 (PMC2712093; doi:10.1371/journal.pone.0006315)
Supplement: File S2 — Ramachandran evaluation plots for models of human eEF1A1 and eEF1A2. Top: Ramachandran plot scores for eEF1A1: most favored regions: 93.6%; additional allowed regions: 5.1%; generously allowed regions: 1.1%; disallowed regions: 0.3%. Bottom: Ramachandran plot scores for eEF1A2: most favored regions: 92%; additional allowed regions: 6.7%; generously allowed regions: 1.3%; disallowed regions: 0%. Ideally, one would hope to have>90% residues in the “most favored” regions of the Ramachandran plot [1], [2]. (0.09 MB PDF) [file pone.0006315.s002.pdf]

Supplementary file S2: Ramachandran evaluation plots for models of human eEF1A1 and eEF1A2.

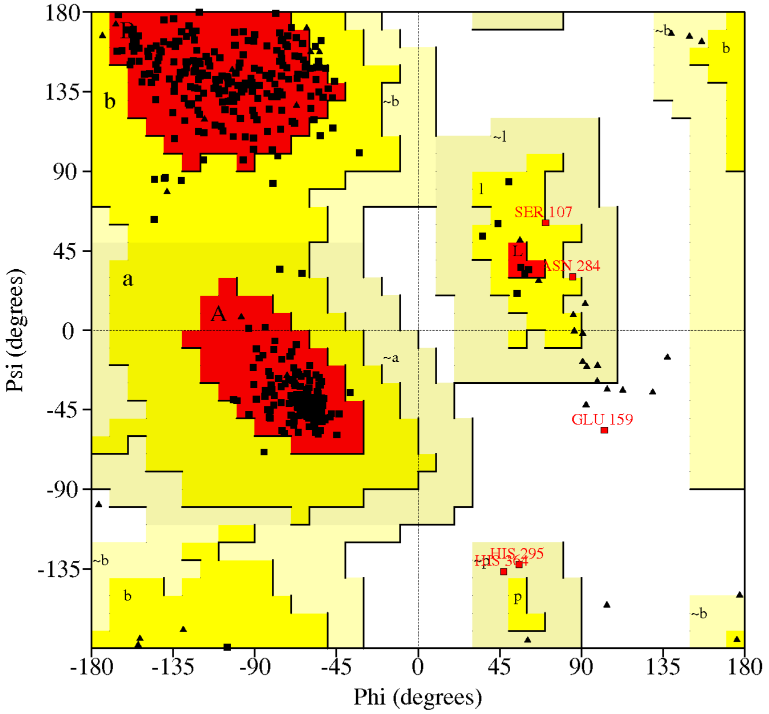

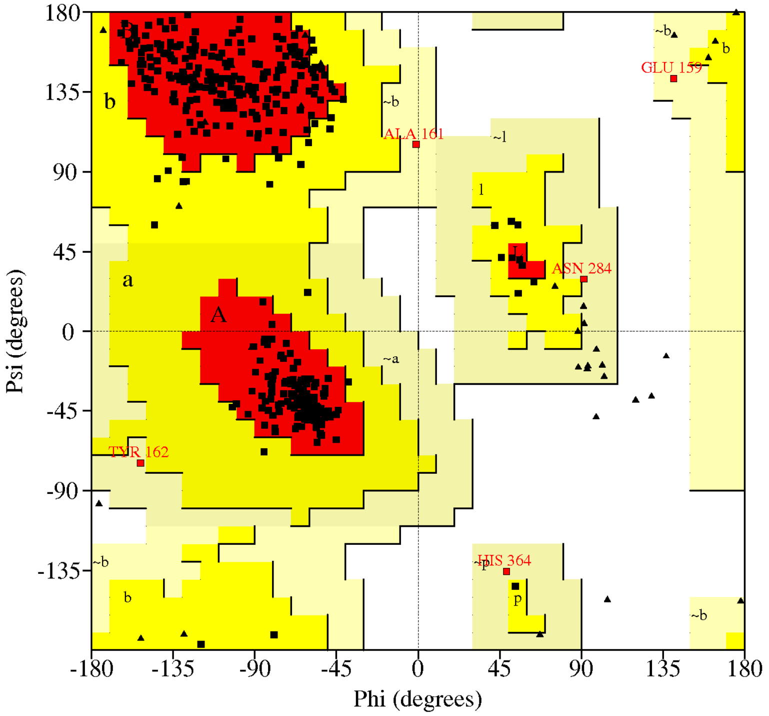

Top: Ramachandran plot scores for eEF1A1: most favored regions: 93.6%; additional allowed regions: 5.1%; generously allowed regions: 1.1%; disallowed regions: 0.3%.

Bottom: Ramachandran plot scores for eEF1A2: most favored regions: 92%; additional allowed regions: 6.7%; generously allowed regions: 1.3%; disallowed regions: 0%. Ideally, one would hope to have >90% residues in the "most favored" regions of the Ramachandran plot [1,2].

References:

1. Morris AL, MacArthur MW, Hutchinson EG, Thornton JM (1992) Stereochemical quality of protein structure coordinates. Proteins 12: 345-364.
2. Laskowski RA, Macarthur MW, Moss DS, Thornton JM (1993) Procheck - a Program to Check the Stereochemical Quality of Protein Structures. Journal of Applied Crystallography 26: 283-291.
