# Supplementary material for: Structural Models of Human eEF1A1 and eEF1A2 Reveal Two Distinct Surface Clusters of Sequence Variation and Potential Differences in Phosphorylation
Source: PLoS One. 2009 Jul 28;4(7):e6315. doi: 10.1371/journal.pone.0006315 (PMC2712093; doi:10.1371/journal.pone.0006315)
Supplement: File S4 — Sequence alignment between archaeal EF1A (Sulfolobus solfataricus) and yeast (Saccharomyces cerevisiae) eEF1A. Pair-wise sequence alignment between eEF1A from yeast vs. EF1A from archaea. From the structures of yeast and archaea, residues involved in domain-domain contacts are depicted as follows: * = yeast amino acid residue involved in domain-domain contact, when bound to eEF1Bα; & = archaea amino acid residue involved in domain-domain contact, free of eEF1Bα; underlined residues = not present in the crystal structures. Identical positions in the alignment are shown with a yellow background; variable positions involved in a domain-domain contact are highlighted with a red background. A total of 42 residues are involved in domain-domain contacts in the eEF1Bα-bound yeast structure, 17 are non-identical at the equivalent position in the archaeal sequence, and conversely, there are 40 residues involved in domain-domain contacts within the unbound archaeal structure, 14 of which are non-identical in the yeast sequence. (0.08 MB PDF) [file pone.0006315.s004.pdf]

Supplementary file S4: Sequence alignment between archaeal EF1A (*Sulfolobus solfataricus*) and yeast (*Saccharomyces cerevisiae*) eEF1A.

```
eEF1A_YEAST       MGKEKSHINVVVIGHVDSGKSTTTGHLIYKCGGIDKRTIEKFEKEAAELGKGSFKYAWVL
EF1A_ARCHAEA      -MSQKPHLNLIVIGHVDHGKSTLVGRLLMDRGFIDEKTVKEAEEAAKKLGKESEKFAFLL

                                                     *  **  *
eEF1A_YEAST       DKLKAERERGITIDIALWKFETPKYQVTVIDAPGHRDFIKNMITGTSQADCAILIIAGGV
EF1A_ARCHAEA      DRLKEERERGVTINLTFMRFETKKYFFTIIDAPGHRDFVKNMITGASQADAAILVVSAKK
                                                   &   &&  &&    &

                                   **   *                                   **
eEF1A_YEAST       GEFEAGISKDGQTREHALLAFTLGVRQLIVAVNKMDSVK--WDESRFQEIVKETSNFIKK
EF1A_ARCHAEA      GEYEAGMSVEGQTREHIILAKTMGLDQLIVAVNKMDLTEPPYDEKRYKEIVDQVSKFMRS
                                &

                  *                                                          *
eEF1A_YEAST       VGYNPKTVPFVPISGWNGDNMIEATTNAPWYKGWEKETKAGVVKGKTLLEAIDAIEQPSR
EF1A_ARCHAEA      YGFNTNKVRFVPVVAPSGDNITHKSENMKWYNG------------PTLEEYLDQLELPPK
                                                                             &

                   * ** * **                 *             *
eEF1A_YEAST       PTDKPLRLPLQDVYKIGGIGTVPVGRVETGVIKPGMVVTFAPAGVTTEVKSVEMHHEQLE
EF1A_ARCHAEA      PVDKPLRIPIQDVYSISGVGTVPVGRVESGVLKVGDKIVFMPAGKVGEVRSIETHHTKMD
                   &&&& & &&                              &&

                                        * *      *  *         * *
eEF1A_YEAST       QGVPGDNVGFNVKNVSVKEIRRGNVCGDAKNDPPKGCASFNATVIVLNHPGQISAGYSPV
EF1A_ARCHAEA      KAEPGDNIGFNVRGVEKKDIKRGDVVGHP-NNPPTVADEFTARIIVVWHPTALANGYTPV
                                        & &         &&        &

                   * **               **                          * * *   **
eEF1A_YEAST       LDCHTAHIACRFDELLEKNDRRSGKKLEDHPKFLKSGDAALVKFVPSKPMCVEAFSEYPP
EF1A_ARCHAEA      LHVHTASVACRVSELVSKLDPRTGQEAEKNPQFLKQGDVAIVKFKPIKPLCVEKYNEFPP
                     &&             & &&  &             &         & &&& & &&

                  * * *     **  *       *
eEF1A_YEAST       LGRFAVRDMRQTVAVGVIKSVDKTEKAAKVTKAAQKAAKK
EF1A_ARCHAEA      LGRFAMRDMGKTVGVGIIVDVKP----AKVEIK-------
                  & &           & &
```

Pair-wise sequence alignment between eEF1A from yeast vs. EF1A from archaea. From the structures of yeast and archaea, residues involved in domain-domain contacts are depicted as follows: ***** = yeast amino acid residue involved in domain-domain contact, when bound to eEF1Bα; **&** = archaea amino acid residue involved in domain-domain contact, free of eEF1Bα; underlined residues = not present in the crystal structures. Identical positions in the alignment are shown with a yellow background; variable

positions involved in a domain-domain contact are highlighted with a red background. A total of 42 residues are involved in domain-domain contacts in the eEF1Bα-bound yeast structure, 17 are non-identical at the equivalent position in the archaeal sequence, and conversely, there are 40 residues involved in domain-domain contacts within the unbound archaeal structure, 14 of which are non-identical in the yeast sequence.
