# Supplementary material for: Structural Models of Human eEF1A1 and eEF1A2 Reveal Two Distinct Surface Clusters of Sequence Variation and Potential Differences in Phosphorylation
Source: PLoS One. 2009 Jul 28;4(7):e6315. doi: 10.1371/journal.pone.0006315 (PMC2712093; doi:10.1371/journal.pone.0006315)
Supplement: File S5 — Guanine-binding pocket in yeast and human variants. Close-up equivalent views of residues involved in binding of GTP/GDP: the yeast template (top left); all six yeast eEF1A structures superposed (top right) and colored differently (see their corresponding PDB IDs below); human eEF1A1 (bottom left); and human eEF1A2 (bottom right). Labeled residues include: Gly19, Lys20, Ser21, Thr22, Asn153, Lys154, Asp156 [GTP/GDP-binding residues] and Asn195 (yeast), Asn/His197 (human eEF1A1/eEF1A2) that show presence of H-bond (indicated by a yellow dashed line and distance in Angstroms) with Asp156, absent in human eEF1A2. Atomic color scheme for yeast template and human variants: nitrogen: blue; oxygen: red; carbon: yellow (note: for clarity hydrogen atoms not shown). (0.15 MB PDF) [file pone.0006315.s005.pdf]

Supplementary file S5: Guanine-binding pocket in yeast and human variants.

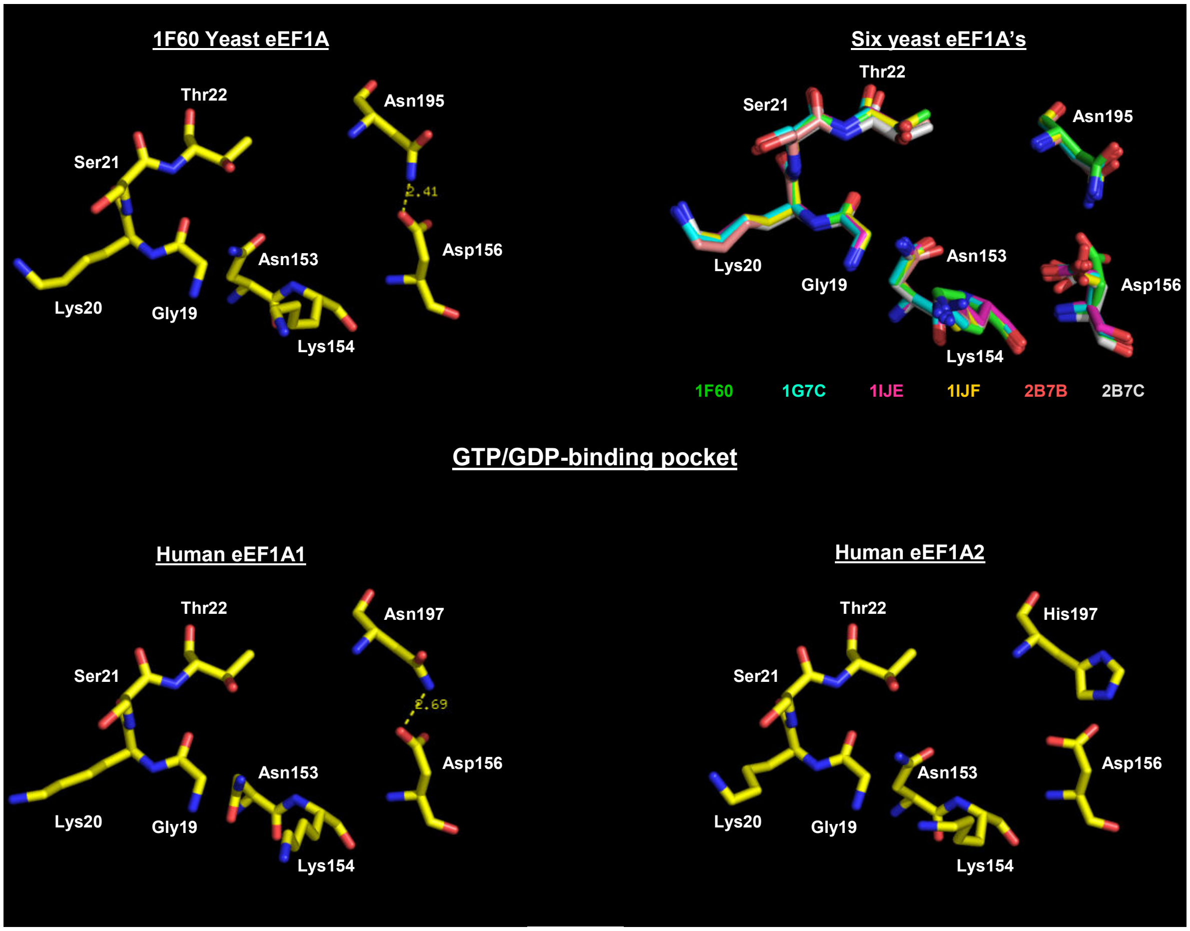

Close-up equivalent views of residues involved in binding of GTP/GDP: the yeast template (top left); all six yeast eEF1A structures superposed (top right) and colored differently (see their corresponding PDB IDs below); human eEF1A1 (bottom left); and human eEF1A2 (bottom right). Labeled residues include: Gly19, Lys20, Ser21, Thr22, Asn153, Lys154, Asp156 [GTP/GDP-binding residues] and Asn195 (yeast), Asn/His197 (human eEF1A1/eEF1A2) that show presence of H-bond (indicated by a yellow dashed line and distance in Å) with Asp156, absent in human eEF1A2. Atomic color scheme for yeast template and human variants: nitrogen: blue; oxygen: red; carbon: yellow (note: for clarity hydrogen atoms not shown).
