# Supplementary material for: Structural Models of Human eEF1A1 and eEF1A2 Reveal Two Distinct Surface Clusters of Sequence Variation and Potential Differences in Phosphorylation
Source: PLoS One. 2009 Jul 28;4(7):e6315. doi: 10.1371/journal.pone.0006315 (PMC2712093; doi:10.1371/journal.pone.0006315)
Supplement: File S6 — Location of known and variable potential phosphorylatable residues on eEF1A1 and eEF1A2 models. Cartoon schematic representation of the 3-D models of eEF1A1 and eEF1A2 depicting the location of the known (orange) and potential (green) phospho-Ser, -Thr and -Tyr residues (side-chain shown in stick representation without hydrogen atoms, for clarity). Potentially phosphorylated Ser445 in eEF1A2 is located in a disordered region, and is hence not seen in the figure. Note: all experimentally confirmed phosphorylation sites are conserved between the human variants, and have not been unambiguously determined specifically to each or both variants. For purposes of the figure, phosphorylated residues are depicted on the models as reported in the PhosphoSitePlus database (http://www.phosphosite.org) [1]: for eEF1A1 (Tyr29, Tyr85, Tyr86, Tyr141, Tyr162, Ser163, and Thr432); and for eEF1A2 (Tyr29, Tyr141, and Tyr254). (0.54 MB PDF) [file pone.0006315.s006.pdf]

Supplementary file S6: Location of known and variable potential phosphorylatable residues on eEF1A1 and eEF1A2 models.

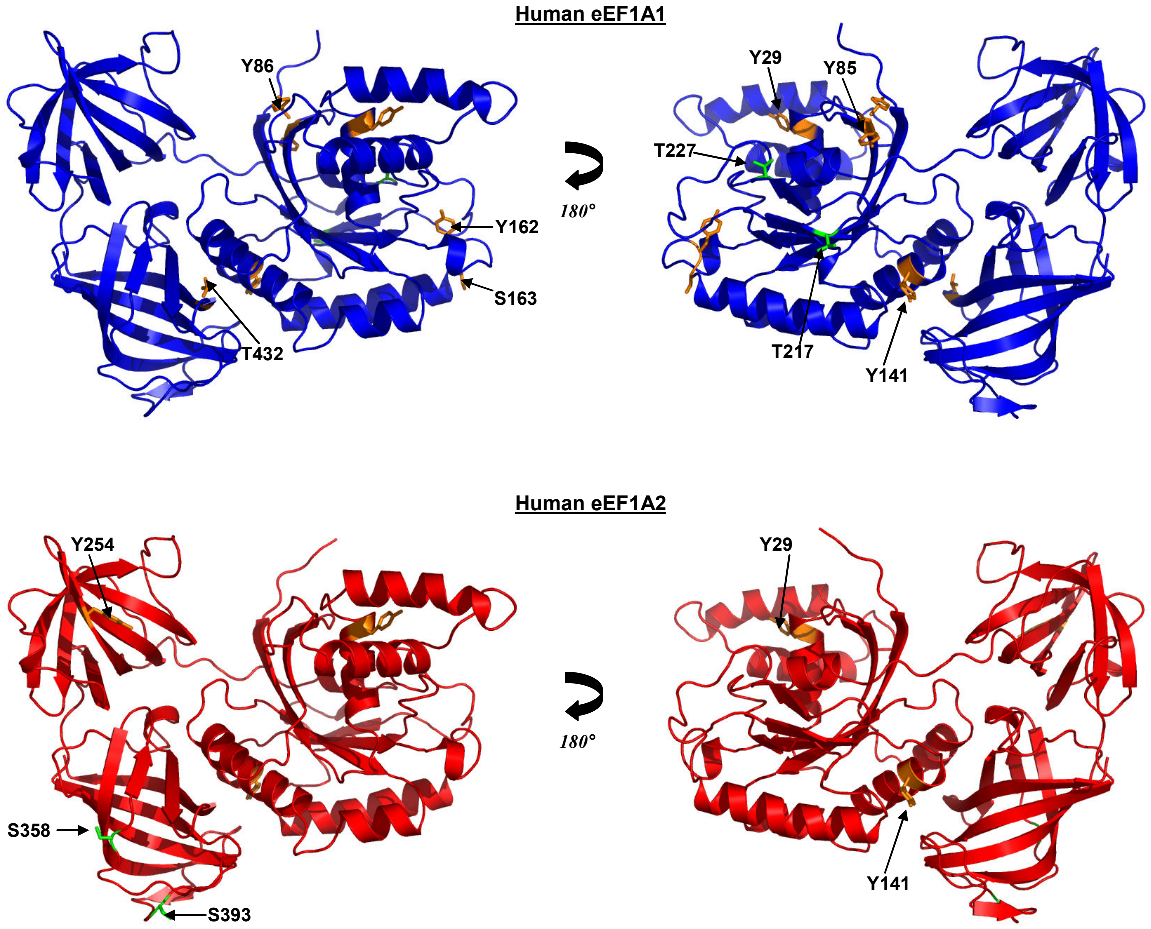

Cartoon schematic representation of the 3-D models of eEF1A1 and eEF1A2 depicting the location of the known (orange) and potential (green) phospho-Ser, -Thr and -Tyr residues (side-chain shown in stick representation without hydrogen atoms, for clarity). Potentially phosphorylated Ser445 in eEF1A2 is located in a disordered region, and is hence not seen in the figure. Note: all experimentally confirmed phosphorylation sites are conserved between the human variants, and have not been unambiguously determined specifically to each or both variants. For purposes of the figure, phosphorylated residues are depicted on the models as reported in the PhosphoSitePlus™ database (http://www.phosphosite.org) [1]: for eEF1A1 (Tyr29, Tyr85, Tyr86, Tyr141, Tyr162, Ser163, and Thr432); and for eEF1A2 (Tyr29, Tyr141, and Tyr254).

References:

1. Hornbeck PV, Chabra I, Kornhauser JM, Skrzypek E, Zhang B (2004) PhosphoSite: A bioinformatics resource dedicated to physiological protein phosphorylation. Proteomics 4: 1551-1561.
